# Enhancing deep-learning training for phase identification in powder X-ray diffractograms

**DOI:** 10.1107/S2052252521002402

**Published:** 2021-04-01

**Authors:** Jan Schuetzke, Alexander Benedix, Ralf Mikut, Markus Reischl

**Affiliations:** a Institute for Automation and Applied Informatics, Karlsruhe Institute of Technology, Germany; b Bruker AXS GmbH, Karlsruhe, Germany

**Keywords:** X-ray diffraction, computational modelling, phase identification, multiphase, deep learning, convolutional neural networks

## Abstract

Within the domain of analyzing powder X-ray diffraction (XRD) scans, manual examination of the recorded data is still the most popular method, but it requires some expertise and is time consuming. The usual workflow for the phase-identification task involves software for searching databases of known compounds and matching lists of *d* spacings and related intensities to the measured data. Most automated approaches apply some iterative procedure for the search/match process but fail to be generally reliable yet without the manual validation step of an expert. Recent advances in the field of machine and deep learning have led to the development of algorithms for use with diffraction patterns and are producing promising results in some applications. A limitation, however, is that thousands of training samples are required for the model to achieve a reliable performance and not enough measured samples are available. Accordingly, a framework for the efficient generation of thousands of synthetic XRD scans is presented which considers typical effects in realistic measurements and thus simulates realistic patterns for the training of machine- or deep-learning models. The generated data set can be applied to any machine- or deep-learning structure as training data so that the models learn to analyze measured XRD data based on synthetic diffraction patterns. Consequently, we train a convolutional neural network with the simulated diffraction patterns for application with iron ores or cements compounds and prove robustness against varying unit-cell parameters, preferred orientation and crystallite size in synthetic, as well as measured, XRD scans.

## Abstract 2

A framework is described for the efficient and realistic simulation of X-ray diffraction scans to train machine- or deep-learning models like convolutional neural networks for the automatic phase-identification task in multiphase compounds.

## 1. Introduction   

Naturally occurring materials, such as ores, usually consist of multiple phases with distinct mass fractions. The composition defines the physical and chemical properties of the substance; thus, an important task for the analysis of the respective materials is the identification and quantification of the present phases. One of the most commonly used analysis methods for crystalline compounds is powder X-ray diffraction (XRD), which utilizes Bragg’s law for measuring a characteristic diffraction pattern and drawing conclusions about the underlying electron-density map and atomic model. For powder samples, the resulting compound pattern is a weighted superposition of all comprised phases, so the measured peaks (interference maxima) can be assigned to the phases with their respective share of the total mixture determined by the peak intensity. The most prevalent technique for measuring diffraction patterns is the Bragg–Brentano focusing geometry which projects the underlying three-dimensional electron density onto a one-dimensional signal, describing the measured intensity as a function of the Bragg angle 2θ (Pecharsky & Zavalij, 2005 ▸).

**Figure 1:**
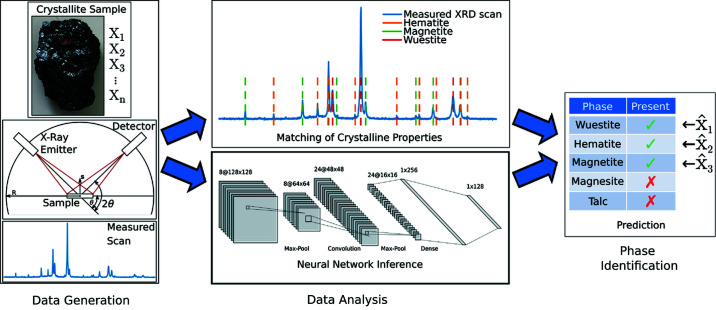
Distinction between the conventional approach and an automatic deep-learning-based analysis for powder XRD scans. Based on pulverized samples of crystalline materials, one-dimensional diffraction patterns are measured using the Bragg–Brentano focusing geometry (left). Typically, the scan is analyzed manually by matching the measured intensity peaks with crystallite properties of reference phases from a database (middle top). Alternatively, a neural-network model is applied to identify the comprised phases in the measured material (middle bottom). Both approaches come to a binary prediction of the comprised phases, while the conventional approach requires the manual intervention of an expert user (right).

The typical workflow for the identification of phases in unknown materials is shown in Fig. 1 ▸. First, a one-dimensional signal is measured for a multiphase crystalline sample using the Bragg–Brentano geometry (Fig. 1 ▸, left). Then, specialized software, like *DIFFRAC.EVA* (Bruker AXS, 2019 ▸) or *Match!* (Putz & Brandenburg, 2014 ▸), is applied, which mostly employs proprietary algorithms for the determination of candidate phases. This algorithm requires a database, such as the ICDD PDF (Gates-Rector & Blanton, 2019 ▸) or the COD (Gražulis *et al.*, 2012 ▸), which stores measured and theoretical patterns or the structural information of known phases. The software analyzes the diffraction pattern for peak positions and intensities and searches the database for phases with matching properties, resulting in a list of proposed candidates from which an expert picks the correct phases. Crystallographic defects, as well as varying lattice parameters owing to solid solution, influence the resulting diffraction pattern and cause a deviation from the database reference. Therefore, an expert is required for estimating whether the degree of discrepancy is still plausible and to make the final pick of the correct phases from the list of proposed candidates (Fig. 1 ▸, middle top and right). Owing to the manual intervention, this process is quite time consuming (Oviedo *et al.*, 2019 ▸), and additionally the results of different users may vary for complex materials as different decisions are made based on varying expertise in evaluating and selecting phases from the proposed candidates.

Alternatively, a machine- or deep-learning model like a neural network can be applied for the data analysis (Fig. 1 ▸, middle bottom). The advantage of machine and deep algorithms is that the phase-identification rules are learned based on training data. Therefore, it is not necessary to describe all possible assessment criteria by algorithms, which would be an extensive task owing to the required expertise for selection of the correct phases. The output of the neural network after inference of the measured scan is a binary prediction (present/absent) of the comprised phases, identical to the conventional analysis step. Here, however, the manual intervention of an expert is not required, resulting in an objective prediction of phases with the ability to predict hundreds of scans in a matter of seconds.

In application, highly specialized machine-learning algorithms, such as the non-negative matrix factorization (NMF) (Long *et al.*, 2009 ▸) approach, as well as neural-network structures (Park *et al.*, 2017 ▸) have already demonstrated good performance for the automatic analysis of XRD data. While the NMF approach learns to describe the diffraction pattern as a linear combination of phases with different fractions, neural networks interpret the diffraction scans as one-dimensional images and detect phases based on intensities under certain 2θ angles (Oviedo *et al.*, 2019 ▸; Wang *et al.*, 2020 ▸). Previous works show that machine- and deep-learning models can be applied for space-group, extinction-group and crystal-system prediction (Oviedo *et al.*, 2019 ▸; Park *et al.*, 2017 ▸), and the assignment of diffraction patterns to their respective phases (Long *et al.*, 2009 ▸; Wang *et al.*, 2020 ▸; Stanev *et al.*, 2018 ▸). Recently, Lee *et al.* (2020 ▸) developed the first neural-network model to identify up to three phases that occur proportionally in a mixture (*e.g.* an ore).

However, measured and labelled XRD scans are not generally publicly available, hence diffraction patterns are simulated to train the automatic models. In order to generate the synthetic XRD scans, the previous works either used specialized frameworks that systematically simulated the diffraction patterns for a specific use case [*e.g.* the ternary system Al–Li–Fe (Bras *et al.*, 2014 ▸)] or exported replicated scans directly from one of the databases which contain information about the crystalline properties of the materials (Oviedo *et al.*, 2019 ▸; Park *et al.*, 2017 ▸; Lee *et al.*, 2020 ▸), such as the Inorganic Crystal Structure Database (ICSD) (Zagorac *et al.*, 2019 ▸). Thus, to apply the models trained with simulated data to real measurements, it is important that all typical effects that occur in XRD scans are also reflected in the artificial training data.

Table 1 ▸ classifies the most typical properties, parameters and effects that influence the diffraction peaks in terms of their position, intensity and shape, with respect to their cause: crystal structure, specimen property and instrument parameters. Stanev *et al.* (2018 ▸) discovered that the NMF reacts sensitively to altered unit-cell parameters compared with training data and consequently modified the specialized NMF algorithm to be more robust against such variations. Under certain circumstances, the developed models may no longer recognize the corresponding phase owing to a small shift in the nanometre range, since the position of the reflection peak changes (according to Bragg’s law). Since neural-network structures cannot simply be designed to be more robust against such diffraction-pattern deviations, it is therefore essential to represent the possible changes in the training data. However, most works represent their phases by a single set of unit-cell parameters and only include variations of background and signal noise, effects which are caused by the measurement itself and not the properties of the phases. Only Lee *et al.* (2020 ▸) represent each of their phases with a multitude of diffraction patterns and also change the peak shape by varying parameters, but are limited in their approach to exported simulated diffraction patterns of the ICSD, which only allows one to modify the anode material (radiation wavelength) and the parameters for the empirical peak-shape function.

As a result, the models developed so far work well for the applications defined in the respective work but are not necessarily transferable to real XRD measurements of all kinds. While several approaches rely on the empirical pseudo-Voigt profile for simulating the peak shape (Park *et al.*, 2017 ▸; Oviedo *et al.*, 2019 ▸; Lee *et al.*, 2020 ▸), some programs [*e.g.* *DIFFRAC.TOPAS* (Coelho, 2018 ▸)] offer a more realistic simulation via the fundamental-parameters approach (Cheary & Coelho, 1992 ▸). Furthermore, so far mainly the offset of peak positions is reflected in the simulated training data, while the influence of diverging peak shape and intensities is still unclear. In addition, phase identification in multiphase compounds has only been tested for relatively simple materials with up to three phases and weight percentages (wps) greater than 10%, restrictions which do not necessarily apply for materials of all kind (Muwanguzi *et al.*, 2012 ▸).

Accordingly, we propose for the first time a framework for the simulation of large XRD scan data sets that enables the variation of peak position, shapes and intensities, and thus generates more realistic diffraction patterns for the purpose of training phase-identification models. Using our framework, we generate single-phase XRD scans with variations for the unit cells, crystallite sizes (CSs) and preferred orientations (POs), and subsequently mix them into multiphase compound samples containing noise and background. With the acquired data we are able to investigate to what extent the previous restrictions in terms of minimum wp and number of phases per mixture affect the performance of the models and how to choose the simulation parameters to obtain ideal results for the phase-identification task. Furthermore, we systematically evaluate the minimum number of training samples required for the models to learn satisfying generalizations, hence reducing the computational efforts and optimizing training times. Finally, we examine the influence of peak-position, shape and intensity variations in order to assess which of the effects is most important to represent in the synthetic data, and thus formulate a recommendation for the optimal simulation of XRD scans for training sets.

## 2. Methods   

### 2.1. Overview   

**Figure 2:**
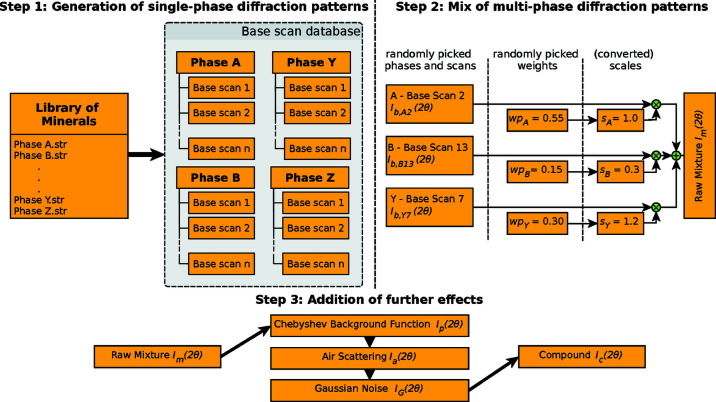
Our synthetic XRD scan generation framework. Based on a library of minerals, a database of base scans with variations of unit-cell parameters, PO and CS is generated. Then, we mix the single-phase diffraction patterns into a multiphase mixture using randomly picked phases and base scans that we scale according to their also randomly assigned weight percentages, wp_*i*_. Finally, we add a polynomial background function, air scattering and noise to complete the fully simulated compound XRD scan.

For utilizing synthetic XRD scans as training data, the classifier needs to know which phases are comprised in each mixture sample (training target). Since mixture scans can be decomposed into a weighted superposition of the diffraction patterns of the comprised phases, we base our framework on single-phase patterns that we subsequently combine into mixtures and add additional effects like background and noise afterwards. Thus, we know exactly which phases are present in the simulated mixtures and their respective fractions. The additional effects are required so that the automatic approach is able to learn a general representation that is robust against all occurring influences. Consequently, we split our XRD scan simulation framework into three steps, as demonstrated in Fig. 2 ▸, and give full details about each level in the respective subsections: Section 2.2, generation of single-phase diffraction patterns[Sec sec2.2]; Section 2.3, mix of multiphase diffraction patterns[Sec sec2.3]; and Section 2.4, addition of further effects (background and noise).

### 2.2. Generation of single-phase diffraction patterns   

Before we start with the generation of synthetic XRD scans, we first limit the number of phases in the library of minerals (in comparison with Fig. 2 ▸). Instead of training one classification model on the identification task of thousands of phases, we identify an application package that contains a subset of related phases that are typically comprised in the same kind of material and train one classifier per application package to improve the performance of the automatic phase identification. We also do not differentiate between minor variations of minerals caused by solid solution and other effects but rather aggregate them into one representative with possible variations of intensities and lattices per mineral.

After putting together a library of minerals, we define which variations are represented in the scans, according to Table 1 ▸. Since we aim to train a classifier for the phase-identification task, it is sufficient for the model to learn in which range the variations are plausible. Accordingly, we choose the most significant entry of each peak-manipulation group (position, intensity and shape) to be varied in our training data. Therefore, we vary (i) the unit-cell parameters to create small differences in the peak positions, (ii) the PO for diverging peak intensities and (iii) the CS to broaden or narrow the peak shape, during the generation of synthetic diffraction scans. In addition, we consider other important parameters, such as the radiation properties, beam conditioning, axial divergence of the beam and the instrument geometry/configuration, by including them in our simulations with static values, but do not vary them between simulated diffraction patterns (We do not consider the absorption, porosity, sample alignment, polarization, crystallinity, disorders, defects, strain and stress because of limitations in our simulation tool.).

Accordingly, we present values for our static parameters, as well as value ranges for all variable parameters, that are used for the generation of synthetic XRD scans in Table 2 ▸. We choose a Cu *K*α emission profile that is restricted to the α_1_ component, which accounts for a monochromator in the experimental setup, and use primary and secondary Soller slits with a 4° angle to suppress the axial beam divergence. For the peak-shape function, we apply an instrumental-based convolution with parameters from the Bruker D2 Phaser. (Details about the D2 Phaser parameters can be found in the example script in the Supporting information.) Furthermore, we simulate all diffraction patterns between 5 and 70° with Δ2θ steps of 0.01°, resulting in 6500 data points per scan. The atomic parameters are strictly defined by the database entries for each phase and are not altered during the generation procedures.

For the second part of Table 2 ▸, which describes the variable parameters for our single-phase diffraction patterns, we base the phase-specific parameters on database entries from the TOPAS structure database. This structure database includes so-called structure files (str) for crystalline phases that contain typical information like unit-cell and atomic parameters. In contrast to other databases, this structure database features one file per mineral and specifies a plausible value range for the parameters and properties, instead of multiple entries per phase. Therefore, only one file per phase has to be processed rather than parsing and combining multiple entries to build the value range. Furthermore, this structure database is to our knowledge the only database that contains information about the *hkl* plane for the March–Dollase correction (PO).

Therefore, we use the specified value ranges provided by the str files to vary the unit-cell parameters, the PO and the CS. Consequently, we simulate the diffraction patterns by drawing from the defined intervals while keeping the constraints of different unit-cell types (*e.g.* a cubic cell requires *a*, *b* and *c* to have the same length, with angles fixed at 90°). Unfortunately, our information source does not contain further information about the plate/needle shaping of the phases (March–Dollase parameter of <1 or >1), so we generally vary all phases between 0.5 and 1.5. (We are aware that this does not exactly reflect reality but need to include a variation for peak intensities.) Eventually, the CS is chosen randomly between 50 and 500 nm for each diffraction pattern and phase.

The calculation of a diffraction pattern is performed by *DIFFRAC.TOPAS* (Coelho, 2018 ▸), which is typically used for the refinement analysis of measured scans, where a simulated pattern is refined to the actual measurement to determine the previously defined scan parameters from Table 1 ▸. Here, we use *TOPAS* ‘in reverse’ and simulate realistic XRD scans by specifying and varying the refinement parameters. Additionally, it features a scriptable interface to automate the generation of large numbers of scans. Alternatively, there are other tools for the simulation of XRD scans available, such as *PowderCell* (Kraus & Nolze, 1998 ▸), *Mercury* (Macrae *et al.*, 2020 ▸) and more, but we employ *TOPAS* as it allows us to specify all the parameters introduced in Table 2 ▸ and we can generate a large number of scans via scripts. An example *TOPAS* script can be found in the Supporting information.

Ultimately, we generate a variation of diffraction patterns for each phase based on the library of minerals using *TOPAS*. The simulated XRD scans, with varying unit-cell parameters, POs and CSs, and other parameters from Table 2 ▸, are henceforth referred to as the ‘base scans’ and form the base-scan database, as demonstrated in Fig. 2 ▸. Each base scan is represented by an ASCII formatted *xy* file with 2θ–intensity pairs and the phases are arranged by folders containing the respective *xy* files. Thus, the intensity under angle 2θ for the base scan *b* of phase *i* is described by *I*_*b,i*_(2θ).

### 2.3. Mix of multiphase diffraction patterns   

In theory, *TOPAS* can be used directly for the simulation of mixture scans, including background and noise. However, it is a resource-heavy program and thus has to be implemented efficiently for the generation of large data sets. Hence, we only use it to simulate the single-phase scans (base scans), and we model the internal algorithms of *TOPAS* for mixing phases and generating noise and background externally. Therefore, we form mixtures by linearly combining generated base scans, based on the *TOPAS* mixing algorithm. (We use it in modified form; the full simplification can be found in the Supporting information.) Typically, the proportion of the phase *i* in the mixture is described by its weight percentage wp_*i*_, with the mass fractions of all comprised phases adding up to 1. Consequently, the scaling factor *s*_*i*_ for each diffraction pattern in the linear combination is directly related to the wp_*i*_ of the corresponding phase.

In detail, we calculate the scaling factor *s*_*i*_ of phase *i* using 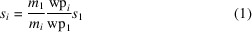
with 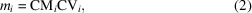
which uses the first phase to put all others in proportion. We choose the first phase randomly when we pick the phases for the mixture, set *s*_1_ to be 1 and calculate the scaling factors of the other phases accordingly (the order of the randomly picked phases does not matter). The parameter *m*_*i*_ expresses the relation of unit-cell mass 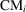 and volume 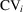, since the peaks are scaled according to the volume of the occurring phase, but in the analysis the proportional mass of the total mixture is usually more important. The values for unit-cell mass and volume depend on the contained atoms and lattice lengths and can be obtained as an output of *TOPAS* when simulating the base scans. Simply put, equation (1) relates the unit cells and wps to calculate the scaling factor in relation to a reference phase.

Thus, the intensities of the mixture *m* can be calculated by addition of the scaled base scans, using 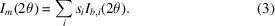



### 2.4. Addition of further effects   

As a final step, background and noise are added. Previous works extracted these components from measured signals (Wang *et al.*, 2020 ▸) but we model the background using a high-order polynomial function (Chebyshev) and uncertainties of the measurement are represented by Gaussian noise, the same as in the work of Lee *et al.* (2020 ▸). The inclusion of Gaussian noise in the simulated scans is necessary so that the network is able to distinguish between diffraction peaks and noise when applied to measured data. Here, we also include effects like air scattering, which occurs at low 2θ angles if the measurement is not performed in a vacuum, that we describe by 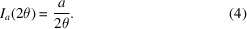
We choose variable *a* such that the measured effect of air scattering is between 0 and 10% of the maximum peak. For the Chebyshev function, we choose a third- to fifth-order polynomial with random coefficients that are constrained to prevent negative intensities from being induced by the background function. With the high-order Chebyshev polynomial, we are able to simulate backgrounds of different kinds, including functions that imitate amorphous content. Finally, we add up the intensities and obtain the complete XRD scan of the compound *c*, 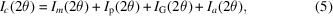
with *I*_p_(2θ) for the polynomial function (Chebyshev), *I*_G_(2θ) for the Gaussian noise and *I*_*a*_(2θ) for possible air-scattering effects.

**Figure 3:**
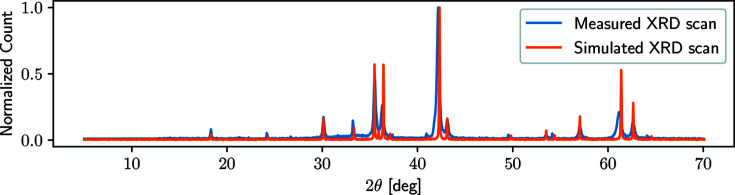
The original measured XRD scan (in blue) in comparison with a simulated scan (in orange), using our framework. Using conventional analysis methods, we identified hematite, magnetite and wuestite with 7, 33 and 60% wp_*i*_, respectively. The differences account for unit-cell, PO and CS variations of the used diffraction patterns. We use a normalized *y* axis (count) to compensate for differences of the absolute intensities owing to different measuring times. Thus, small effects like air scattering are almost not noticeable but present.

Finally, we obtain a synthetic XRD scan that can be used for training of a machine-learning model since we know exactly which phases are present with their respective wps and scaling factors. In addition, our framework enables the use of additional effects like background and noise as augmentation methods during the training process. Fig. 3 ▸ visualizes a simulated mixture in direct comparison with its measured counterpart. We reconstruct the actual XRD scan by applying the same wps, but notice small deviations that are caused by varying unit-cell parameters, POs and CSs of the used base scans.

### 2.5. Deep-learning architecture and training procedure   

For training and evaluation of the data sets generated by our framework, we use a simple convolutional-neural-network architecture developed by Lee *et al.* (2020 ▸), which features three convolutional layers in exchange with max-pooling layers and three concluding fully connected layers that apply dropout during training. We adapt the output layer to contain the same number of neurons as we have phases in our application package. Full information and details can be found in the Supporting information. The neural networks are implemented and trained using the *TensorFlow* (Abadi *et al.*, 2015 ▸) and *Keras* (Chollet, 2015 ▸) packages and a NVIDIA Tesla K80 graphics card.

#### 2.5.1. Scaling of the input   

Most importantly, we normalize the simulated XRD scan before we feed it into the network to improve the convergence. Neural networks fine-tune their weights by a backpropagation algorithm and large intensities cause high derivatives, so we scale our input data using a min–max scaling that sets the highest intensity to 1 and the lowest to 0, while the intermediate values are linearly interpolated. As a side effect, this also accounts for differences of absolute intensities owing to diverging measuring times, as we applied and visualized in Fig. 3 ▸. Prior to the normalization, we perform a logarithmic transformation of the scans (often used while manually evaluating scans). This compensates for the differences between high and small peaks and improves the visibility of minor peaks.

#### 2.5.2. Optimizer and loss function   

Since we are trying to solve a phase-identification task, our automatic approach has to predict a binary output (either present or absent) for every candidate phase. Also, multiple phases can be present at the same time, which corresponds to a multi-label classification task, so we employ a binary cross-entropy loss function. In our experiments we found that often the convergence strongly depends on the initial configuration of weights and in some cases the networks did not converge at all, despite using the identical training data and optimizer configuration as in previous runs. Thus, we eventually decided to use the advanced-optimizer and learning-rate-schedule *AdamW* (Loshchilov & Hutter, 2019 ▸) with cosine learning-rate annealing and warm restarts (Loshchilov & Hutter, 2017 ▸). Consequently, we start with a relatively high learning rate that is reduced by cosine annealing and restarts at the high learning rate after some epochs, with the interval between restarts increasing during the training process. Accordingly, the optimizer does not get stuck in local minima and should find a better generalization without the possibility of not converging at all.

#### 2.5.3. Metrics and evaluation   

During the training process, loss gives a general idea about the performance of the network. It is based on the continuous output values of the network (between 0 and 1 owing to the sigmoid activation function of the output layer) in relation to the binary target labels. In discrete terms, a phase is typically counted as predicted present if the output value of its respective neuron is greater than (or equal to) 0.5 and interpreted as absent for smaller than 0.5. Therefore, the prediction of every phase in every sample can be evaluated as true positive (TP), true negative (TN), false positive (FP) and false negative (FN), and consequently the accuracy is calculated as the relation of correctly predicted phases (TP + FN) to the total number of components in the mixtures. However, the accuracy does not tell the whole story about the performance in our use case, since it should be used in applications with an equal number of positives and negatives. In our mixtures, the number of absent phases (negatives) is usually disproportionately greater than the number of present components, so a classifier that predicts every phase as absent (negative) can have a good accuracy metric, despite not working as intended. Consequently, we also calculate the recall, 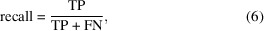
the precision, 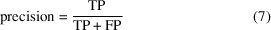
and the *F*1 score, 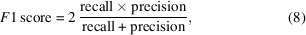
which is the harmonic mean of the two metrics. In comparison with the accuracy, these metrics do not include TNs in their calculations and are therefore better suited for our use case.

### 2.6. Design of experiments   

Subsequently, we need to specify the generation of synthetic data that is particularly suitable for training robust models for the phase-identification task. First, we set some restrictions to reduce the number of parameters for generating training data. Thus, we represent every phase by 50 base scans, assuming that this number is sufficient for a good representation of divergence in single-phase diffraction patterns. Although the variations for some phases are more extensive than for others, for the sake of simplicity we generate the same number of base scans for every phase (50). Furthermore, we set a static 2θ range from 5 to 70° with a Δ2θ step of 0.01° for all diffraction patterns, resulting in 6500 data points per synthetic XRD scan, and scale every XRD scan by a logarithmic transformation and min–max scaling before we feed it into the network. This last constraint is also a restriction of the neural-network structure, since we implement a network with a static number of neurons in the input layer, so each neuron must correspond to the intensity of the same 2θ angle for every training sample.

Table 3 ▸ describes the resulting training and test data sets for our experiment: Series A–Series D. Each training set is used for training of the described neural-network structure, so the difference between model variants is the applied data during the training process. For clarification, each model variant has a specifier related to the experiment series and the training set, like B1 for the first training set of Series B. Additionally, every training set has an associated validation set with 50 000 samples and identical parameters.

We start our experiments with the investigation of the influence of peak position, shape and intensity manipulating parameters in Series A. Real scans contain most or all of the properties and parameters described in Table 1 ▸, therefore we generate a test set with 50 000 samples and variations of all three peak-manipulation groups. As training data we generate eight sets with 100 000 samples each that contain the possible combinations of the three groups (0 to 3 variations present) and examine how well the trained models perform against the test set. From this evaluation we conclude the influence of the peak-manipulation groups on the robustness of the classifier for use with real measurements. Here, the training and test set consist of multiphase samples with five comprised phases per sample and a minimum wp of 2%.

Afterwards, we test in Series B and Series C how robust the classifier is in case the parameters of the training data do not correspond exactly to those of the test data. Previously, Lee *et al.* (2020 ▸) generated 800 000 synthetic scans with three phases per mixture and a minimum wp of 10% per phase for training and testing purpose. Similarly, we generate training data and a test set with identical parameters in series A, but studies on real iron ore samples found that those naturally occurring materials contain any number between 1 and 10 (some even more) phases per sample (Muwanguzi *et al.*, 2012 ▸). Thus, we investigate how to choose the optimal value for the minimum wp in Series B by generating samples with three phases per mixture and wps between 10 and 2% that we test against samples with a mass fraction per phase of at least 1%. Subsequently, we set the minimum wp to 2% and generate mixtures with 3 to 7 phases in Series C to determine the required number of phases per sample, so the trained classifier works well for mixtures of all kinds.

Finally, we evaluate in Series D the importance of data-set-scope size, which is heavily limited by the hardware components. A training set with 500 000 generated samples for the previously set 2θ range with 6500 points results in 25 GB required memory space (with float64 datatype). By dividing the training samples into batches, the training process uses less concurrent space but the generation and loading process of the entire training set demands optimized routines. In the previous experiment series, we generated all training sets with 100 000 samples, which suggests that performance could be improved with more training data. Therefore, we train our networks with up to 500 000 training samples to determine the required amount of synthetic XRD scans.

In order to verify the validity of the results, we run all four experiment series with two different applications. As introduced in Section 2.2, we limit the TOPAS structure database to a set of phases typically present in iron ore samples with 28 possible minerals and, secondly, we select a more complex application package with 76 cement phases. While the iron ores set features phases that mostly differ significantly from each other, the cements phases include candidates that are hard to distinguish, like Dolomite ordered versus disordered and Brownmillerite variants CaFeAlO versus CaFeMgSiO. Here, we look at structures that are very similar, like elements of a substitutional solid-solution series, or just the replacement of ions in a certain structure type that fit by size and charge. They exhibit the same crystal structure with only small variations of the lattice parameters and, consequently, very similar *d* spacings. As the substitution also does not affect the scattering power much, the related intensity variation is not conclusive. This makes it difficult, if not impossible, even for an expert, to distinguish such species solely based on the diffraction signal. Consequently, the combination of XRD with a chemical analysis becomes mandatory to differentiate between some candidates. As a result, we expect the neural networks to perform worse for the cements set owing to the higher complexity.

## 3. Results   

### 3.1. Overview   

Using our framework, we determine a parameter set for the training of robust phase-identification algorithms. Accordingly, we divide the results section into four subsections, according to the experiment series of Table 3 ▸. In most of our evaluations we train one classifier three times with each of the different training sets to reduce the variations of the optimization algorithm. Thus, we visualize our results using bar charts for the mean *F*1 scores of the models plus error bars for the minimum and maximum scores. For the data-set-scope evaluation we utilize a line chart to demonstrate performance progression for increasing numbers of training samples. Additionally, as well as analyzing the metrics for the test data sets as a whole, we also break them further down and report on suitable subsets of the test data to give more detailed information about the performance of the classifiers trained with different training data sets.

### 3.2. Base-scan variation effects   

First, we investigate which variations in the training data set cause the classifiers to learn a robust generalization against the possible influences. For this purpose, we test classifiers trained with the possible combinations of changes in unit-cell parameters, PO and CS against a data set containing all possible variations. Additionally, we test the performance of a model that does not contain any variation in the training data set (No Variation), representing each phase with only one base scan.

**Figure 4:**
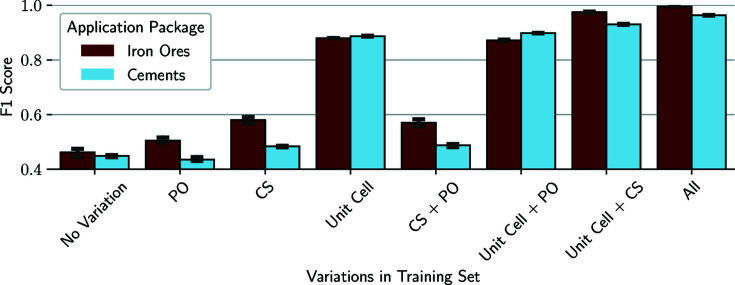
*F*1 scores of the Series A test sets for classifiers trained with no variation in the training set, only PO, CS or unit-cell parameters varied, and the other possible combinations of these. We report the scores for the iron ore package (dark red) and the cements (light blue).

Fig. 4 ▸ shows the *F*1 score for the possible variations in the training data set. Here, we achieve the lowest scores overall for classifiers trained with no variation at all, signifying that it is definitely relevant for the phase-identification task to represent the possible influences in synthetic training data. In general, the variation of unit-cell parameters leads to the largest improvement with a plus of over 40%, while diverging CSs and PO causes only small enhancements in the performance, even in combination. Additionally, the inclusion of unit-cell variations leads to small divergences between the three runs for each model variant, as shown by the small error bars.

Interestingly, in the case of cements, the mere variation of the PO leads to the classifiers performing worse than the baseline models, which were trained without varying any parameters. This is owing to the solid-solution variants contained in the package, which can only be distinguished from each other by small differences in intensity ratios of peaks. By varying the intensities alone, the mixed crystals are more difficult to differentiate as a result, while the classification of the remaining phases does not improve significantly. When comparing the impact of CS and PO, the former appears to be slightly more important for training a robust model, as indicated by the better performance of the respective models (baseline to PO/CS, unit-cell variation + PO/CS). For the best possible performance, however, it is important to include variations in the peak intensities, since the models trained with all possible variations perform best.

**Figure 5:**
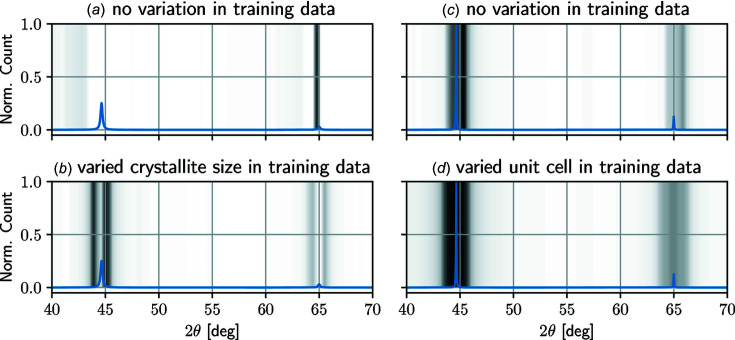
CAMs for a base scan of iron alpha of different classifier variants. The heatmap from white (not important) to black (highly important) visualizes the regions of the input scan that are most relevant for the predictions of the classifiers. For (*a*) and (*c*), the model was trained without variation in the training data, while (*b*) includes CS variation in the training data and (*d*) includes unit-cell variations. In the left column, the base scan of iron alpha is simulated using a small CS (50 nm), and in the right column the base scan features a varied unit cell.

For a better understanding of the trained neural networks, we visualize the class activation maps (CAMs), which highlight the regions of the input scan that are most relevant for the prediction of the model. Fig. 5 ▸ shows the CAMs of different model variants for base scans of iron alpha with a heatmap ranging from white (no importance) to black (highly important). In the left column, Fig. 5 ▸ displays a base scan with ∼50 nm CS and the CAMs for a model (*a*) trained with no variation and a model (*b*) trained with data that reflects the possible variation in CSs. Here we observe that model (*a*) trained without peak-shape variation does not recognize the first diffraction peak (no grey bars around the peak at ∼45°), while model (*b*) correctly identifies both peaks and consequently outputs the correct phase.

In the right column of Fig. 5 ▸, we illustrate the CAMs of iron-alpha base scans for a model (*c*) without variations in a training set versus a model (*d*) trained with unit-cell varying base scans. Since the above model (*c*) never learns that lattice parameters, and thus diffraction 2θ angles, are variable, the model predicts iron alpha based on a narrow range of angles. In comparison, the fully connected classification layers of model (*d*) learn to accept diffraction peaks from a broader range of angles to predict the phase. Overall, the variation of parameters is necessary for fine-tuning the fully connected layers of the neural networks, while the CS is also relevant for learning adequate convolution filters.

### 3.3. Evaluation of minimum weight percentage   

**Figure 6:**
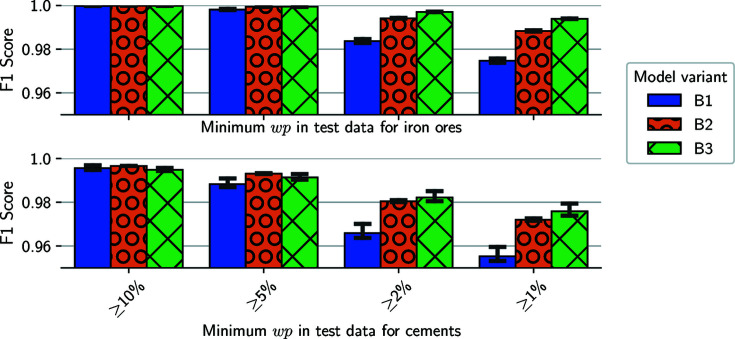
The evaluation of three classifier variants, trained with mixtures, which consist of three phases with minimum wps of 10 (B1), 5 (B2) and 2% (B3). We report the *F*1 scores for a test set with a minimum wp of 1% for the three comprised phases plus subsets of greater minimum wps. Each bar visualizes the mean of three training runs with error bars indicating the minimum and maximum values.

For the determination of an optimal minimum wp value, we train classifiers with the three training sets of Series B, consisting of mixtures with a wp equal to or greater than 10, 5 or 2%, resulting in three classifier variants (B1, B2 and B3, respectively). Afterwards we apply the trained variants to the designated test set with down to 1% wp. Additionally, we determine the prediction scores for subsets of the testing set that only consist of mixtures with 10, 5 and 2% minimum wp. Fig. 6 ▸ shows the *F*1 scores of the three classifier variants B1–B3 for the subsets and the full test set (≥1%) for our two application packages (iron ores with 28 phases and cements with 76).

In our first subset that only consists of mixtures with three phases and mass fractions greater than 10%, we report *F*1 scores of nearly 100% for all three model variants applied to the iron ore case. Additionally, the scores are only slightly worse for the cements package where we also report *F*1 scores of at least 99% for the first subset. With decreasing minimum wps, the *F*1 scores across all classifier variants deteriorate. While the scores decline slowly for the classifier B3, the performance of the B1 classifiers decreases more quickly. However, it should be noted that all classifiers are able to identify phases with smaller wps than those in their training sets (to a certain degree). Thus, it is possible to apply a neural-network model trained with mixtures of phases with a wp greater than or equal to 5% (B2) for the phase-identification task of all wps (down to 1%) and still achieve a *F*1 score of ∼99% (97% in the cements case). Overall, the models trained with a minimum wp of 2% (B3) perform best on our test sets with three phases present in extremely small ratios.

### 3.4. Phases per mixture   

**Figure 7:**
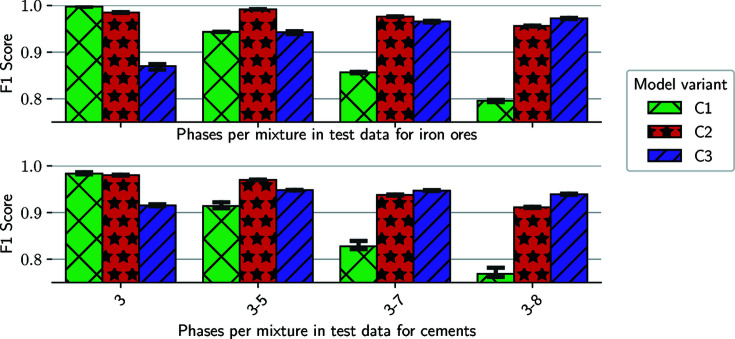
*F*1 scores for the test set of the phases per mixture test series plus error bars for the three runs. Each classifier is trained with a data set that contains either 3 (C1), 5 (C2) or 7 (C3) phases per mixture and tested against compounds of 3–8 phases.

Using model variants C1, C2 and C3, we investigate how many phases are ideally mixed into training-data compounds so that the classifier is subsequently able to analyze mixtures with any number of components. Accordingly, we present the *F*1 scores for the test set of the three classifier variants trained with mixtures of 3, 5 and 7 phases, respectively, in Fig. 7 ▸. Additionally, we report the performances for subsets of the whole test set with mixtures consisting of only 3, 3 to 5 and 3 to 7 phases, in addition to the overall test set with 3 to 8 phases per mixture. [The first classifier (C1) is also identical to B3, both are trained with three phases per mixture and a minimum wp of 2%.]

The results show similar behaviour like the previous evaluation, with an overall decrease in performance for increasing numbers of phases per mixture and the classifiers performing best for the subset of parameters that are identical to their training sets (in most cases). Here, however, the decline of the performance is more significant for the C1 classifier when applied to more sophisticated mixtures, with a relative difference of ∼20% for both application packages. Also, the C3 variants perform significantly worse on compounds consisting of just three phases in comparison with the other two classifiers. Interestingly, the network trained with five phases per training sample (C2) achieves a higher *F*1 score for the iron ores and 3–7 phases subset than the matching variant (C3). While the wp analysis showed that variant B3 is the most stable variant with the highest overall scores, here, the network trained with five phases per mixture (C2) achieves the most consistent results across all test subsets.

### 3.5. Data-set scope   

**Figure 8:**
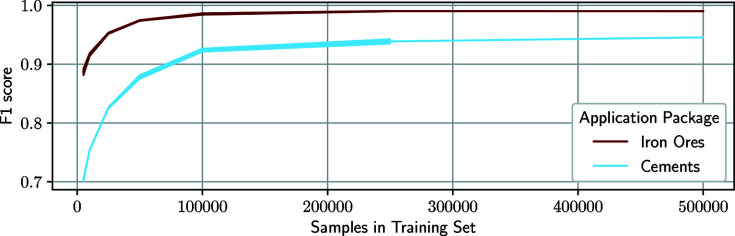
The progression of *F*1 scores for classifiers of iron ores (dark red) and cements (light blue) depending on the scope of the training data set (Series D). We range the training data sets between 5000 and 500 000 samples.

Lastly, we review how many training-data samples the models require in order to achieve the best possible generalization for XRD data. Thus, we train models with training data sets containing between 5000 and 500 000 synthetic samples and test them against 50 000 other simulated mixture patterns in the data-set-scope test. Fig. 8 ▸ shows the *F*1 score in relation to the number of samples in the training set for our application packages containing 28 or 76 phases. While the classifiers for the iron ores achieve a score of almost 90% for as little as 5000 training samples, the performance of the cement mixture classifiers starts at 70% for a training with 5000 samples. This is partly owing to the fact that there are more phases present in the cements application package and therefore the probability of each phase being one of the seven random phases per mixture is lower. Consequently, more training samples are required to represent the possible variation of each phase.

Additionally, we analyze how well the networks trained for the cements package are able to differentiate between similar phases, such as the Brownmillerite variants. For a network that is able to perfectly distinguish between all classes, there is no relation between outputs of different classes, while a classifier that struggles to separate two classes is likely to show a correlation between predicted probabilities for both classes. Table 4 ▸ shows the correlation coefficients between the network output for Brownmillerite variants CaFeAlO and CaFeMgSiO and increasing number of samples in the training sets. A higher correlation coefficient indicates that the network is less likely to differentiate between the two similar phases. While the overall *F*1 score improves from ∼70% to ∼82% between 5000 and 25 000 training-set samples, the correlation coefficient does not change too much. Thus we conclude that the networks first focus on the phases that are easier to identify. Once most phases are classified correctly, the networks learn the subtle differences between similar phases using 100 000 training samples and further improve their classification scores. However, given certain unit-cell, CS and PO parameters, it is not possible to distinguish the similar Brownmillerite variants without a chemical analysis, so the networks can never achieve perfect classification scores for all possible variations of these candidates.

In general, Fig. 8 ▸ and Table 4 ▸ show that there is only a marginal performance gain for training sets with more than 100 000 samples, which confirms the choice of the training-data scope for Series A–C. For the iron ore package, the *F*1 score increases by ∼0.1% between 250 000 and 500 000 training samples, while the cements improve by 0.7% for the same numbers. Both cases suggest that there is a performance saturation, which occurs earlier in the case of iron ore mixtures. Generally, it is questionable whether the phase-identification task can be solved perfectly (1.0 score) using an automatic analysis model for application packages of all kinds. While the cement set is composed in such a manner that a perfect prediction based on XRD scans alone is impossible, the networks do not achieve a perfect classification score for the iron ores either. However, owing to the size of the training data set (50 000 synthetic mixtures), it was not possible to determine human performance as a benchmark.

In both cases it is evident that an increase in training data also leads to an improvement in performance. What is not shown in the two graphs, however, is that as the number of training-data values increases so do the training times. For all our models (regardless of application package) the training stopped after approximately the same number of epochs (∼90) but the time per epoch increased linearly. Accordingly, our system trained a model with 100 000 training samples in ∼4 h, while the same model required 20 h to train with 500 000 samples. Thus, it is necessary to consider to what extent a small improvement in accuracy can be justified with an increase in training time.

## 4. Conclusions   

In summary, we present a framework that enables the simulation of XRD scans in large quantities and is therefore suitable for the generation of training data for machine-learning models. Our framework complements existing alternatives for the simulation of diffraction patterns, which represent variation in natural measurements by different unit-cell parameters and peak shapes, with the variance of peak intensities by randomly chosen preferred orientations. Using the presented framework, we investigated how to arrange the most optimal synthetic data set for training a neural network to perform an automatic phase-identification task.

Most importantly, variances of the unit cell, which alter peak positions, influence the neural network most significantly and therefore have to be represented in synthetic training data. Moreover, we discovered that peak-shape and intensity variations by randomly chosen March–Dollase parameters and crystallite sizes lead to an improvement in phase identification, as may result in the real world when samples are not optimally prepared. Hence, variations of all three peak-manipulation groups (position, intensity and shape) are required for training a robust classification network.

Furthermore, we evaluated how to determine training-set parameters like the number of phases per mixture or the minimum wp per phase to achieve the best phase-identification performance across samples of all kinds. In most cases, the model variant with the parameters of the training set matching those of the test sample performed best. However, the exact parameters of the unknown sample are rarely known in advance (*e.g.* how many phases are present), so it is not always possible to apply the model trained with matching parameters. In our experimental setup, the classifiers trained with five phases per mixture and a minimum wp of 2% provided a balanced prediction across mixtures of different complexities. Additionally, we found that ∼100 000 synthetic mixtures are required for sufficiently training a neural network for the phase-identification task of 28 candidate iron ore phases, while the set of 76 phases that may be found in cement samples requires ∼250 000 training samples. For larger training sets we observed a saturation of performance with linearly increasing training times that do not justify the added value.

The next step for our framework is to use the simulated diffraction patterns to compare more machine- and deep-learning algorithms with each other and also include performance scores of an expert to better assess the determined metrics. For the training of neural-network models it is important to reduce the learning rate after each optimizer step (instead of epoch) to utilize greater data-set scopes for a faster and better convergence of the classifiers. One restriction of our framework, however, is that information about the preferred orientation of different minerals is only available for a small number of phases. Thus, an alternative may be to substitute the March–Dollase model with a random scaling of peaks that is not phase dependent. 

## Supplementary Material

Supporting information. DOI: 10.1107/S2052252521002402/fc5051sup1.pdf

## Figures and Tables

**Table 1:** Classification of properties, effects and parameters that influence powder XRD scans Both the position and intensity of the peaks, as well as the peak shape, can be manipulated. These influences can be attributed proportionally to the crystal structure, specimen properties and instrument parameters. Adapted from the work of Pecharsky & Zavalij (2005 ▸).

	Crystal structure	Specimen property	Instrument parameters
Peak position	Unit-cell parameters (*a*, *b*, *c*, α, β, γ)†	Absorption‡	Radiation – wavelength§
		Porosity‡	Instrument/sample alignment‡
			Axial divergence of the beam§
Peak intensity	Atomic parameters (*x*, *y*, *z*, *B*, *etc*.)§	PO†	Geometry and configuration§
		Absorption‡	Radiation – Lorentz polarization‡
		Porosity‡	
Peak shape	Crystallinity‡	Grain size†	Radiation – spectral purity§
	Disorder‡	Strain‡	Geometry§
	Defects‡	Stress‡	Beam conditioning§

† Parameters that vary between scans.

‡ Parameters that we do not consider.

§ Parameters that are static for all scans.

**Table 2:** Values and variations for the defined static and varied parameters in synthetic XRD scans We use a convolution-based profile shape with parameters from a Bruker D2 Phaser, with Cu *K*α_1_ emission profile and Soller slits to reduce axial divergence for all diffraction-pattern simulations.

Parameter name	Value/variation
Static parameters	
Radiation – wavelength (Å)	Cu *K*α profile (1.541)
Radiation – spectral purity	Cu *K*α_1_ only
Axial divergence of beams	Soller slits with 4° angle
Geometry and configuration	Bruker D2 Phaser setup
2θ range and Δ2θ (°)	5 to 70 with Δ0.01
Varied parameters	
Unit-cell parameters	Str-file entries
PO	March–Dollase parameter, 0.5–1.5
CS (nm)	50–500

**Table 3:** Details about the experimental setups for our four testing series concerning number of samples, phases per mixture, and the minimum wps for our training, validation and testing sets The parameters of the validation sets correspond to the series and model variants but feature only 50 000 samples.

		Training set (and validation set)			Test set		
Experiment series	Model variant	Number of samples	Phases per mixture	Minimum wp	Number of samples	Phases per mixture	Minimum wp
Series A	1–8	100 000	5	2	50 000	5	2
		For all possible combinations			With all three variations present		
Series B	1	100 000	3	10	50 000	3	1
	2	100 000	3	5			
	3	100 000	3	2			
Series C	1	100 000	3	2	50 000	3–8	2
	2	100 000	5	2			
	3	100 000	7	2			
Series D	1	5000	7	2	50 000	3–8	2
	2	10 000	7	2			
	3	25 000	7	2			
	4	50 000	7	2			
	5	100 000	7	2			
	6	250 000	7	2			
	7	500 000	7	2			

**Table 4:** Correlation coefficients between network outputs for Brownmillerite variants CaFeAlO and CaFeMgSiO and increasing training-set scopes

Training-set scope	Correlation coefficient
5000	0.307
25 000	0.291
100 000	0.132
250 000	0.131
