# Supplementary material for: Enhancing deep-learning training for phase identification in powder X-ray diffractograms
Source: IUCrJ. 2021 Apr 1;8(Pt 3):408–20. doi: 10.1107/S2052252521002402 (PMC8086162; doi:10.1107/S2052252521002402)
Supplement: Supplementary file 1 [file m-08-00408-sup1.pdf]

IUCrJ

Volume 8 (2021)

Supporting information for article:

Enhancing deep-learning training for phase identification in powder X-ray diffractograms

Jan Schuetzke, Alexander Benedix, Ralf Mikut and Markus Reischl

Appendix

TOPAS defines the scaling factors s_i of a mixture with n phases as follows:

$$s_j = x_j * \sum_{\substack{i=1 \\ i \neq j}}^{n} s_i * m_i, \tag{1}$$

where s_1 is set to 1,

$$m_i = CM_i * CV_i \tag{2}$$

for the Unit Cell Mass CM_i and Unit Cell Volume CV_i of the *i*-th phase and

$$x_n = \frac{wp_n}{m_n * (100 - wp_n)} \tag{3}$$

for the respective weight percentage wp_n. The complexity of solving the system is increasing for higher amounts of phases in the compound but can be simplified in the following way, exemplarily for 3 phases:

$$s_2 = x_2 * (s_1 m_1 + s_3 m_3) \tag{4}$$
$$s_3 = x_3 * (s_1 m_1 + s_2 m_2) \tag{5}$$

with (5) inserted in (4):

$$\begin{aligned} s_2 &= x_2 * (s_1 m_1 + m_3 * (x_3 * (s_1 m_1 + s_2 m_2))) \quad (6)\\ &= s_1 m_1 x_2 + s_1 m_1 x_2 x_3 m_3 + x_2 s_2 m_2 x_3 m_3 \\ s_2 * (1 - x_2 m_2 x_3 m_3) &= s_1 m_1 x_2 * (1 + x_3 m_3) \quad (7) \end{aligned}$$

with

$$\begin{aligned} 1 - x_2 m_2 x_3 m_3 = 1 - \frac{wp_2 wp_3 m_2 m_3}{m_2 m_3 (1 - wp_2)(1 - wp_3)} &= \frac{(1 - wp_2)(1 - wp_3)}{(1 - wp_2)(1 - wp_3)} - \frac{wp_2 wp_3}{(1 - wp_2)(1 - wp_3)} \\ = \frac{1 - wp_2 - wp_3 + wp_2 wp_3 - wp_2 wp_3}{(1 - wp_2)(1 - wp_3)} &= \frac{1 - wp_2 - wp_3}{(1 - wp_2)(1 - wp_3)} \\ = \frac{wp_1}{(1 - wp_2)(1 - wp_3)} \end{aligned}$$

and

$$1 + x_3 m_3 = 1 + \frac{wp_3 m_3}{m_3 (1 - wp_3)} = \frac{(1 - wp_3) - wp_3}{(1 - wp_3)} = \frac{1}{(1 - wp_3)}$$

inserted into (7):

$$\begin{aligned} s_2 * \frac{wp_1}{(1 - wp_2)(1 - wp_3)} &= s_1 m_1 x_2 * \frac{1}{(1 - wp_3)} \\ s_2 &= x_2 * \frac{s_1 m_1}{(1 - wp_3)} * \frac{(1 - wp_2)(1 - wp_3)}{wp_1} \\ s_2 &= \frac{s_1 m_1 wp_2}{m_2 (1 - wp_2)(1 - wp_3)} * \frac{(1 - wp_2)(1 - wp_3)}{wp_1} \\ s_2 &= \frac{m_1}{m_2} * \frac{wp_2}{wp_1} * s_1 \quad (8) \end{aligned}$$

Equation (8) shows that the scaling factor of the second phase is completely unrelated to properties of the third phase and this can be demonstrated analogically for phase three with Equation (5). In conclusion only the relations to the first phase are needed when calculating scaling factors. For compounds with more than three phases, the validity of Equation (8) can be confirmed by a linear solver. Therefore, the scaling factor in relation to the first phase can be calculated for every phase *i* using

$$s_i = \frac{m_1}{m_i} * \frac{wp_i}{wp_1} * s_1. \tag{9}$$

Exemplary TOPAS input script

```
'***** General job parameters *****
iters 0
yobs_eqn !calc_pattern.xy = X;
'***** 2theta range (in degrees) *****
min 5
max 70
del 0.01
'***** 2-column .XY output file  *****
Out_X_Ycalc(iron_alpha_001.xy)
' ***** Instrument geometry and monochromator *****
' ***** D2 Phaser specific *****
Rp 141
Rs 141
LP_Factor( 0)
'***** Linear position sensitive detector *****
lpsd_th2_angular_range_degrees 5
lpsd_equitorial_divergence_degrees 0.3
axial_conv
    filament_length 12
    sample_length 25
    receiving_slit_length 12
    primary_soller_angle 4
    secondary_soller_angle 4
    axial_n_beta 30
'***** Emission profile *****
CuKa1(0.0001)
'ymin_on_ymax -> 0.0001
'***** Copy from structure file (.str) of phase ****
str
phase_name "Iron alpha"
space_group 229
scale sc_iron_alp  0.001
Phase_LAC_1_on_cm( 2378.416225)
Phase_Density_g_on_cm3( 7.865840408)
site s1 num_posns 2 x =0; :  0 y =0; :  0 z =0; :  0 occ FE  1 beq  0.5
'Change value to vary unit cell
Cubic(a_iron_alp 2.867547688)
'Change value to vary crystallite size
CS_L(@, 500)
'Change first 1 to vary march dollarse parameter
PO(, 1,, 1 0 -1)
MVW( 0, 0, 0)
'Macro MVW is filled out automatically in output-file after run (.out)
'required for scaling equation, use unit cell (M)ass and (V)olume values
'***** END OF PYTHON-GENERATED TOPAS INPUT FILE ****
```

Table 1: Architecture of the employed neural network

Number	Layer-Type	Number Neurons	Parameters
0	Input	6500	
1	Convolutional 1D	6500(x64)	kernel-size: 20, filters: 64, stride: 1
2	MaxPooling 1D	2167(x64)	pool-size: 3, stride: 3
3	Convolutional 1D	2167(x64)	kernel-size: 15, filters: 64, stride: 1
4	MaxPooling 1D	723(x64)	pool-size: 2, stride: 3
5	Convolutional 1D	362(x64)	kernel-size: 15, filters: 64, stride: 2
6	MaxPooling 1D	181(x64)	pool-size: 1, stride: 2
7	Flatten	11584	
8	Fully-Connected	2500	dropout-value: 0.5
9	Fully-Connected	1000	dropout-value: 0.5
10	Fully-Connected	28/76	dropout-value: 0.5
11	Output	28/76	
